# A Machining State-Based Approach to Tool Remaining Useful Life Adaptive Prediction

**DOI:** 10.3390/s20236975

**Published:** 2020-12-06

**Authors:** Yiming Li, Xiangmin Meng, Zhongchao Zhang, Guiqiu Song

**Affiliations:** School of Mechanical Engineering & Automation, Northeastern University, Shenyang 110819, China; m.ether0330@gmail.com (X.M.); Brunozhang@yahoo.com (Z.Z.); gqsong@mail.neu.edu.cn (G.S.)

**Keywords:** remaining useful life, LightGBM, loss function, curse of dimensionality, multi-information fusion

## Abstract

The traditional predictive model for remaining useful life predictions cannot achieve adaptiveness, which is one of the main problems of said predictions. This paper proposes a LightGBM-based Remaining useful life (RUL) prediction method which considers the process and machining state. Firstly, a multi-information fusion strategy that can effectively reduce the model error and improve the generalization ability of the model is proposed. Secondly, a preprocessing method for improving the time precision and small-time granularity of feature extraction while avoiding dimensional explosion is proposed. Thirdly, an importance coefficient and a custom loss function related to the process and machining state are proposed. Finally, using the processing data of actual tool life cycle, through five evaluation indexes and 25 sets of contrast experiments, the superiority and effectiveness of the proposed method are verified.

## 1. Introduction

Remaining useful life (RUL) prediction is an important research direction in the field of prognostics and health management (PHM). Especially in modern Computer numerical control (CNC) machining, this tool is a key component of CNC machine tools, and its life management plays an important role in the efficiency of CNC machine tools, workpiece quality and cost control. Therefore, RUL prediction of the tools in the early stage is of great significance. 

In recent years, with the development of sensor technology and signal processing technology, many RUL prediction methods have been put forward one after another. These papers have contributed to sensor technologies, signal processing and decision-making strategies for process monitoring [1,2,3].

The estimation of RUL can be divided into two categories—i.e., physical model-based methods and data-driven methods [4]. Usually, the physical method-based model is a formula derived from failure physics to predict the theoretical damage evolution—e.g., ref [5] proposed a Taylor tool life formula-based method to estimate RUL of the tools. In ref [6,7], the Paris-Erodogan model was used to predict the crack path and crack size of bearings and gearbox pinions, respectively. The authors of [8] proposed a Paris-Erodogan model combined with the finite element model to represent the time evolution of tooth cracks in the gear. In ref [9], a method to extend the processing equation based on Taylor speed to predict the RUL of the tool is proposed.

However, when modeling the degradation process, many practical factors, such as cutting parameters and processing steps, are easily overlooked [10]. This is one of the limitations of Taylor’s tool life formula. Additionally, most of the coefficients involved in the physical model are determined experimentally, which makes it difficult to match the complex industrial production environment.

To solve the above problems, the data-driven method is proposed as an alternative approach. The data-driven methods build models by mining historical data of machinery—e.g., the artificial neural network (ANN) model trained by historical data was used in [11] to predict the RUL of machinery. Luo [12] used multiple sensors to collect information for collaborative merging to provide more accurate perceptions and make further optimal decisions. In ref [13], Luo et al. also proposed that the decision-making process of automated mechanical and electrical systems relies on multiple-sensor data, and the information from distributed sensors needs to be fused in a robust way. In [14], the RUL of CNC machining lathes and the associated confidence limits are estimated by using a dynamic Bayesian network. The authors of [15] proposed a three-stage method for assessing machine health degradation and using Cox’s proportional hazard model and a support vector machine (SVM) to predict RUL based on vibration signal fusion. Loutas [16] used a support vector regression (SVR) to estimate the RUL of rolling bearings and establish a data-driven approach.

Soualhi [17] proposed a method combining Hilbert–Huang Transform (HHT), SVM and SVR to detect bearings and obtain an estimation of RUL by a further time series prediction based on SVR. In ref. [18], a tool wear detection system based on Relevance Vector Machine (RVM) classifiers is constructed, which realized the multi-classification of the tool wear state during cutting. In reference [19], the SVR model is used to predict tool wear. In ref [20], the neuro-fuzzy network is adopted to predict the tool wear and RUL. Lei [21] proposed a model-based method for predicting mechanical RUL. This method used the vibration signals from the accelerated degradation testing dataset of the rolling bearings to prove the accuracy of the proposed model. Wang [22] used an adaptive Expectation-Maximum (EM) algorithm and Bayesian update algorithm to update the parameters of the data-driven model and proved the effectiveness of the proposed method in improving RUL estimation accuracy and shortening convergence time. 

In addition to the abovementioned studies, in ref. [23,24,25,26,27,28,29,30,31,32], there are also many scholars who have conducted relevant research. However, the existing data-driven methods still have some limitations, and the problems that need to be solved can be summarized as follows:

(1) Traditional predictive models cannot achieve self-adaptiveness in different complex processes. A conservative protection strategy could cause excessive tool wear and lead to a rapid increase in cutting force, affecting the processing quality of the workpiece and reducing the yield of the qualified workpiece; excessive protection strategies could waste the RUL of the tool, increase unnecessary downtime and lead to a decrease in production efficiency and an increase in manufacturing costs. Finding RUL prediction methods related to the process will effectively improve the quality of workpiece, increase production efficiency and optimize workpiece costs.
(2) Traditional data sources rely on a single type of data and are mostly single dimensions. Such a prediction model will lack the coupling nonlinear influence factors under a different process, resulting in the reduction in credibility in the prediction process, reduction in the confidence interval, the generalization ability of the model not being strong and the actual working conditions not being able to be accurately described. It is especially important to choose the right data dimensions and combinations.
(3) When extracting small-time granularity features in the traditional way, the features extracted by the quadratic features are directly added to the previously extracted features, which causes the inconsistency of the sample sparsity, and reduces the generalization ability of the model, the over-fitting of the model, “curse of dimensionality” and other issues. It is necessary to find a preprocessing method to solve the dimensional explosion problem.

In order to solve the above problems, this paper proposes a RUL prediction method that considers the related process and processing state. The contributions of this paper include:

(1) A multi-information fusion strategy that can effectively reduce the model error and improve the generalization ability of the model is proposed.
(2) A preprocessing method for improving the time precision and time granularity of feature extraction while avoiding dimensional explosion is proposed.
(3) An importance coefficient and a custom loss function related to process and machining state are proposed. The new prediction model can realize the adaptive prediction of RUL under different processes.

The rest of this paper is organized as follows. Section 2 is a description of the proposed method. Then, the effectiveness of the proposed method is discussed in Section 3. Finally, Section 4 concludes the present work.

## 2. Units Proposed Method

### 2.1. Architecture of the Proposed Method

**Figure 1:**
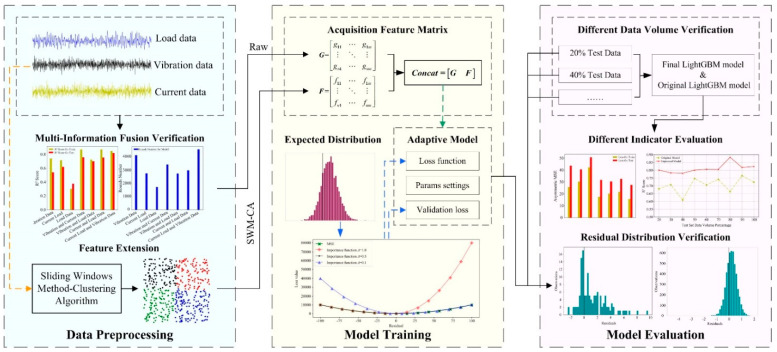
Architecture of remaining useful life (RUL) prediction method based on LightGBM.

This paper presents a LightGBM-based approach to RUL prediction related to process. Figure 1 shows the architecture of the RUL prediction method based on LightGBM, which consists of the following three parts: data preprocessing, model training and model evaluation. In part 1, load data, a combination of different data types of vibration data and current data, verifying the superiority of the multi-information fusion combination method, are used; using the sliding windows method-clustering algorithm to expand features and avoid "curse of dimensionality", the extracted feature matrix and the original feature matrix are merged in the model training phase. In part 2, secondary correction of residuals is made to adjust the distribution to meet the expected requirements of industry, choosing the LightGBM model that meets the fast and timely needs of the industry as the original model. The importance model and the custom loss function associated with the custom coefficient are proposed to adjust the original model. In part 3, different test sample sets are used to verify the new prediction model. (1) Visual analysis by using different quantitative indicators to ensure a more comprehensive assessment of the model’s error, fit and generalization ability. (2) Draw a histogram of the residual distribution, visually observe and evaluate the damage at both sides of the risk from the perspective of engineering significance.

### 2.2. SWM-CA

In the traditional RUL prediction research, the existing literature mostly performs feature extraction in the time domain and frequency domain in a given time interval. However, some transient mutation characteristics of the data within a given time interval may be masked due to larger time being fine-grained, resulting in the inability to capture the characteristics of the abrupt signal, causing imperfect or under-fitting of the model. However, the sliding windows method will generate a large number of subdata segments. The new features extracted from a large number of subdata segments will cause inconsistency in sample sparsity, reduction in model generalization ability and model over-fitting such as the curse of dimensionality problem. Therefore, this paper proposes using the clustering algorithm (CA) to perform unsupervised analysis on all subsamples after sliding windows method (SWM) segmentation and to prevent feature redundancy caused by improved resolution while ensuring small-time granularity, preventing feature redundancy due to increased resolution and making the features perform well in the model.

Unsupervised learning is used to classify the intrinsic properties and laws of data. The clustering algorithm divides the sample set $D=\left\{x_{1},x_{2},\ldots ,x_{m}\right\}$ into a number of disjoint subsets, namely the sample cluster $C=\left\{C_{1},C_{2},\ldots ,C_{k}\right\}$, and minimizes the squared error of the divided sample clusters [33].

(1) $$E=\sum_{i=1}^{k}\sum_{x\in C_{i}}{\left\|x-\mu_{i}\right\|}_{2}^{2}$$

where ***μ**_i_* is the mean vector of the sample cluster *C_i_*, $\mu_{i}=\frac{1}{\left|C_{i}\right|}\sum_{x\in C_{i}}x$.

The effect of clustering is weighed by introducing the intra-cluster similarity DB index and the inter-cluster similarity Jaccard coefficient. 

The Davies–Bouldin Index (DB index) measures internal indicators for clustering performance,

(2) $$\mathrm{DBI}=\frac{1}{k}\sum_{i=1}^{k}\underset{j\neq i}{\max}\left(\frac{avg(C_{i})+avg(C_{j})}{d_{cen}(C_{i},C_{j})}\right)$$

where $d_{cen}()$ is used to calculate the distance between two samples, $d_{cen}(C_{i},C_{j})=dist(\mu_{i},\mu_{j})$; avg(C) represents the average distance between samples within cluster *C*. 

The Jaccard Coefficient is an external indicator for clustering performance measurement.

(3) $$\mathrm{JC}=\frac{a}{a+b+c}$$

(4) $$\left\{ \begin{array}{l} a=\left|SS\right|,SS=\left\{(x_{i},x_{j})|\lambda_{i}=\lambda_{j},\lambda_{i}^{*}=\lambda_{j}^{*},i<j\right\}\\ b=\left|SD\right|,SD=\left\{(x_{i},x_{j})|\lambda_{i}=\lambda_{j},\lambda_{i}^{*}\neq \lambda_{j}^{*},i<j\right\}\\ c=\left|DS\right|,DS=\left\{(x_{i},x_{j})|\lambda_{i}\neq \lambda_{j},\lambda_{i}^{*}=\lambda_{j}^{*},i<j\right\}\\ d=\left|DD\right|,DD=\left\{(x_{i},x_{j})|\lambda_{i}\neq \lambda_{j},\lambda_{i}^{*}\neq \lambda_{j}^{*},i<j\right\} \end{array}\right.$$

where set *a* represents a sample pair that belongs to the same cluster in *C* and belongs to the same cluster in C*; set *b* represents a sample pair that belongs to the same cluster in *C* and is not affiliated with the same cluster in C*; set *c* indicates that the sample pairs that are not affiliated to the same cluster in *C* and belong to the same cluster in C*; set *d* represents a sample pair that is not affiliated with the same cluster in *C* and is not affiliated with the same cluster in C*.

The sliding windows method clustering algorithm (SWM-CA) has the following advantages: by using the SWM-CA to process data, it can achieve fine-grained features of small-time coverage in small-time, and it can also eliminate a large number of redundant features in extended features, so as to alleviate the feature loss and redundancy caused by the improvement of time resolution.

### 2.3. Loss Function of p-LightGBM

(1) The importance coefficient

When solving the actual problem, if our predicted RUL is shorter than the actual RUL, then the tool will be replaced before it expires, wasting the service life of the tool and increasing the using cost; if the predicted RUL is longer than the actual RUL, then the tool will continue to work in the failed state for a considerable period of time, causing the workpiece to fail or even be directly scrapped. The positive and negative values of the residual between the true value of the RUL and the predicted value are often not equivalent. Ideally, we hope that the prediction model can accurately predict the RUL, but in reality, the error is unavoidable. Therefore, the residual distribution needs to be developed in the direction of actual expectation, and the risk is secondarily controlled by correcting the residual distribution. Therefore, in the face of this kind of unequal risk, this paper proposes the importance coefficient p, which evaluates the workpieces and tools under different processes and adjusts and distinguishes the punishments on both sides of the risk to achieve model self-adaptively and predictions of the RUL under different processes.

First, in order to quantify the importance of the process steps, this paper proposes the importance coefficient p related to the process and machining state. The importance coefficient p can be calculated not only by the method mentioned in this article, but also by other methods. Different importance determination strategies can be used to quantify the importance, and a simple way is used in this paper.

(5) $$p=\frac{T_{sp}}{T_{sp}+\Delta }$$

where *p* is importance coefficient; *T_sp_* is the sequence of the process in the whole process and the value is the actual processing process scaled to the interval [0, 1]; *Δ* is the machining state coefficient, and its value is the reciprocal of the importance factor of the previous process or the current process— the value is in the range of (0, 1). 

**Figure 2:**
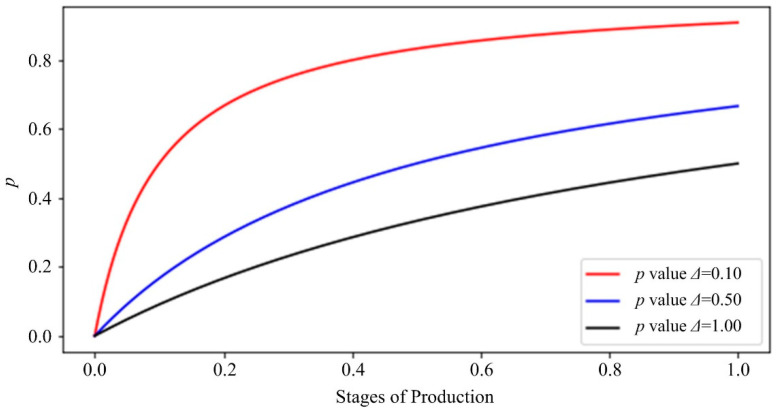
Importance coefficient curve of different processing state coefficients.

In order to more intuitively observe the influential rule of the processing state coefficient on the importance coefficient, several representative machining state values were selected to visually describe them, and the machining state coefficients *Δ* = 0.1, *Δ* = 0.5 and *Δ* = 1.0 were used, respectively. The importance of the process in different cases, in turn, indicates the three cases in which the preorder or current machining process is important, general and unimportant. Figure 2 shows the importance coefficient curve of different processing state coefficients. It can be seen that if the importance of the preprocessing process is high, that is, *Δ* has a small value, the importance coefficient *p* can ensure that a large weight is given in the premachining state, and can make sure that harsher penalties are imposed on the negative residuals when the preorder or current processing steps are highly important.

(2) Custom loss function

In order to achieve adaptive adjustment of the unbalanced sides of the risk, and to match the industrial production practice, this paper proposes a custom loss function (CLF) considering the importance coefficient *p*. We take the CLF as an example and transform it adaptively so that the model can be coupled with the actual situation of the industry.

(6) $$\left\{ \begin{array}{cc} \frac{\delta }{1+e^{-1/\left(p-1/2\right)}}(\left|\alpha \right|-\frac{1}{2}\delta ), & \alpha \geq \delta \\ \frac{\delta }{1+e^{-1/\left(p-1/2\right)}}\alpha^{2}, & 0\leq \alpha <\delta \\ (1-\frac{\delta }{1+e^{-1/\left(p-1/2\right)}})\alpha^{2}, & -\delta <\alpha <0\\ (\delta -\frac{\delta }{1+e^{-1/\left(p-1/2\right)}})(\left|\alpha \right|-\frac{1}{2}\delta ), & \alpha \leq -\delta \end{array}\right.$$

where *α* is the residual; *δ* is a parameter of CLF used to enhance the robustness of the squared error loss to outliers. That is, when the residual is smaller than *δ*, the square error is used, and when the predicted value is larger than *δ*, the linear error is used.

**Figure 3:**
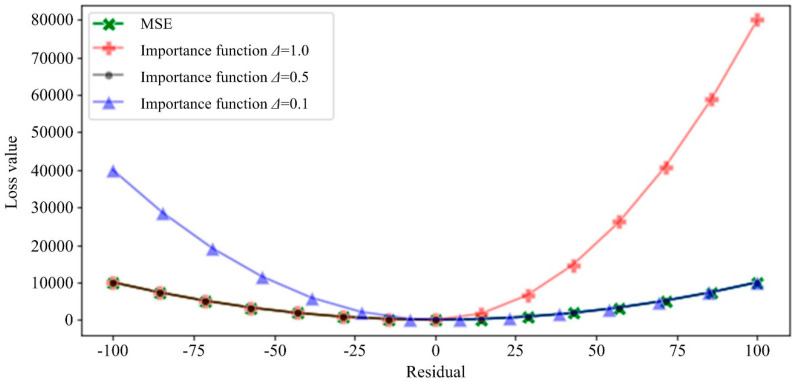
Comparison of importance function and MSE function of different processing state coefficients when *T_sp_* = 0.5.

In order to visually verify and reflect the penalty effect of CLF in different situations on both sides of the risk, CLF corresponding to different importance degrees was compared with the Mean Square Error (MSE). The figure below was used so that when we set different importance parameters and the residuals are in different positive and negative directions, the loss function imposes different degrees of punishment on the model, as shown in Figure 3.

The level of punishment for the positive and negative residuals was judged by observing the magnitude of the first derivative of the loss function on both sides of the positive and negative residuals. In Figure 2 and Figure 3, it can be seen that when the current process is in the middle of the overall process (*T_sp_* = 0.5), the preorder steps are important (*Δ* = 0.1), general (*Δ* = 0.5) and not important (*Δ* = 1.0). In the three cases, the corresponding importance of *p* are 0.83, 0.5 and 0.33, respectively. When *p* = 0.5, the custom function degenerates to the equivalent of the penalty on both sides, and the effect is the same as the normal loss function. In the case of *p* = 0.5, we applied different levels of punishment to the positive and negative residuals, and the effect is in line with the expected hypothesis.

(3) Verify the engineering practice effect of CLF

**Figure 4:**
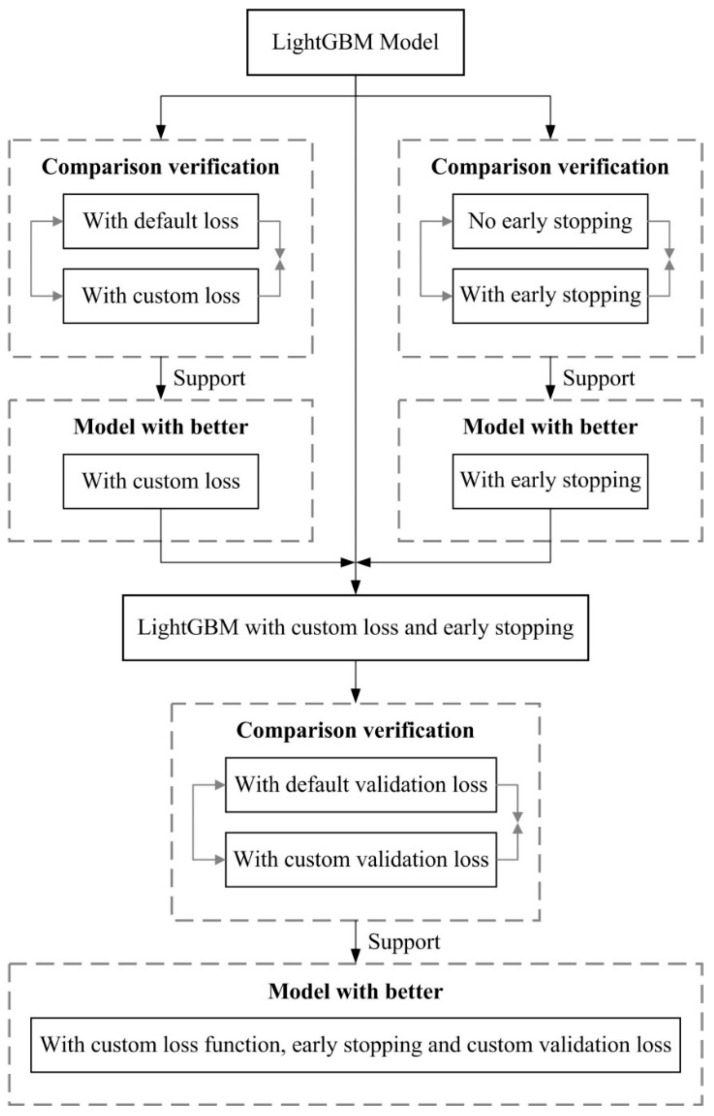
The flowchart of verification comparison.

In order to verify the self-adaptiveness of the proposed CLF, five groups of comparative engineering practice are conducted in this section. We used the LightGBM model for training [34]. LightGBM is a Gradient Boosting Regression Tree (GBDT) model based on the histogram algorithm in the Boosting framework. LightGBM can speed up and optimize computing memory and efficiency. LightGBM uses two ways to achieve its fast accuracy, namely gradient-based one side sampling (Goss) and exclusive feature bundling (EFB). Because such advantages are extremely important for large-scale data, we chose this model as the target model. Figure 4 shows the flowchart of verification. Table 1 is the model fit conditions under different comparisons.

**Figure 5:**
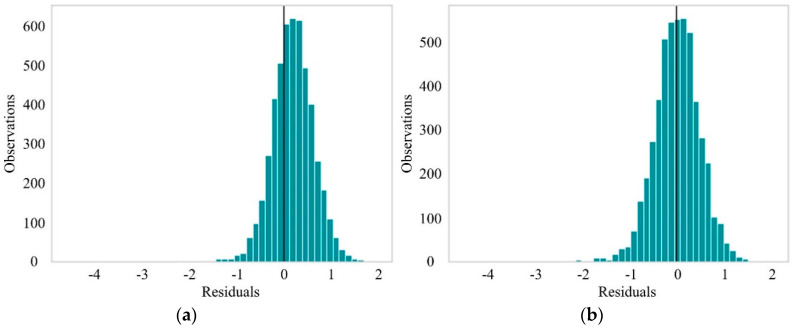
Error histograms of predictions from different loss functions (*Δ* = 0.1). (**a**) LightGBM with early stopping, custom loss and custom validation loss. (**b**) default LightGBM.

The comparison validation in Table 1 was designed to verify the effectiveness using the following three aspects: the CLF, the early stopping hyperparameter and an external validation function which matches the CLF. First, under the condition of fixed Boosting Rounds, the asymmetric loss (train) values of default LightGBM and LightGBM with custom loss were 0.628296 and 0.27638, LightGBM with custom loss exhibited a better performance. At the same time, LightGBM with custom loss performs equally well in more important test sets, effectively improving the prediction accuracy of the model. Second, LightGBM with an early stop had better loss convergence and a better generalization ability than default LightGBM. Its test set had a 38% reduction in asymmetric loss and was particularly effective. Using LightGBM with early stop and custom loss can also optimize the purpose of improvement. Last, the model was optimized using a custom external validation function. The effect is more obvious when compared to functions that do not use custom external validation. The model using the custom validation function reaches the optimal value when training 241 rounds, and the one without using it reaches the optimal value in the 1848 round. In the case where both are optimal at the same time, the model generalization ability using the custom verification loss was better, and its performance in the test set improved by 12%. The result shows that: (1) LightGBM with custom loss has a smaller error of convergence value; (2) using the custom loss function and matching the loss verification function as the external verification loss, the obtained model has better adaptability and robustness, and has faster error convergence speed. To further illustrate this, we observed the residual histogram (Figure 5) for more details.

We selected the training samples with the preorder process importance *Δ* = 0.1 for training, and according to the previous theory, we must have more severe penalties for dealing with negative residuals. As can be seen from Figure 5a, the LightGBM model considering the custom loss made more predictions on the right side of the error histograms, and the residual shifted to the right—namely, the actual value was greater than the predicted value. It is further proved that using the custom loss function proposed in this paper can effectively reduce the loss in the opposite direction to the industrial expectation and make a second correction to the loss in order to obtain a lower hazard in practice.

## 3. Experiments and Discussion

In this section, the proposed method is verified by five evaluation indexes and 25 groups of comparative experiments. Experiments in Section 3.2, Section 3.3 and Section 3.4 were conducted to verify the effectiveness of multi-information fusion strategy and data type combination, SWM-CA and RUL prediction models.

### 3.1. Process Description

**Figure 6:**
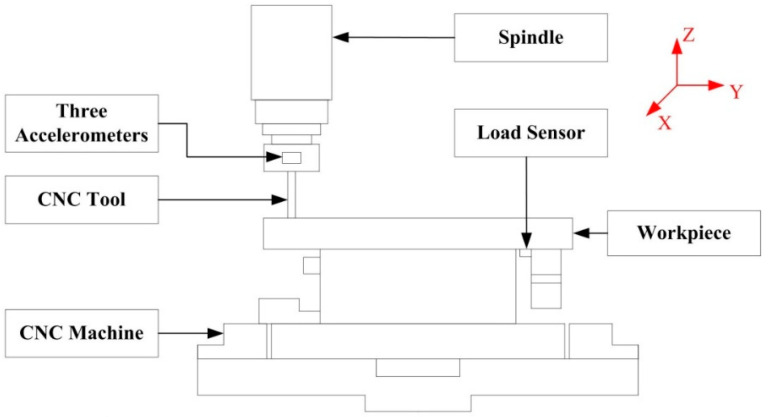
The main components and sensor positions of the Computer numerical control (CNC).

The data used in the experiments were collected from a CNC machining process. This process is a full-life cycle cutting experiment using a ball-end mill. Since the experimental process is to collect the complete life cycle of multiple sets of tools as soon as possible, we simplified the processing process. Although the simplified process will be different from the real industrial production, the data obtained in this way can still be used for data analysis and theoretical verification. Throughout the experiment, the data collects controller signals, process information and sensor data during processing according to the Cyber Physical Systems (CPS) framework. Altogether 745 min, 24.78 GB and 149 valid data samples were collected. Using the full-life data of the nine ball-end mills as the basis for building the dataset. We separated the data from the two ball-end milling cutters as part of the generalization ability of the final test model. This part of the data does not participate in any training of the model. In the experiment, we collected three kinds of signals: load signal, vibration signal and current signal. In addition, in the vibration signal and current signal, we collected three-dimensional vibration signal data, which are x, y, z direction and three-phase current data. Figure 6 shows the main components and sensor positions of the CNC.

### 3.2. Verifying the Validity Muti-Information Fusion Strategy and Data Type Combination

Ref. [35,36,37] proved that the degradation of the tool can be estimated by the current signal/electric power of the machine tool; ref. [38,39] used the spindle load as a data source to predict the RUL of the tool; ref. [40,41,42,43,44,45] used vibration signals as the basis of the data-driven model to achieve RUL prediction. The above studies are based on a single data type. In order to verify the superiority of the proposed multi-information fusion strategy and the validity of the data type combination, this paper used the LightGBM model to compare the prediction results of engineering practice with seven different types and different combinations of data types. Since this paper is trying to emphasize the idea of feature extraction in the time dimension and the integration of the model with engineering practice, it has not expanded and extended more features. Therefore, we selected some representative features as basic features and input them into the model. Feature sets including time domain, frequency domain and time–frequency features were considered, as shown in Table 2. 

This focuses on asymmetric loss to compare the error sizes and generalization capabilities of the model. The R^2^ Score in statistics, which is the goodness of fit, was introduced for normalization comparison. Table 3 is the prediction result of different types and different combinations of data types.

**Figure 7:**
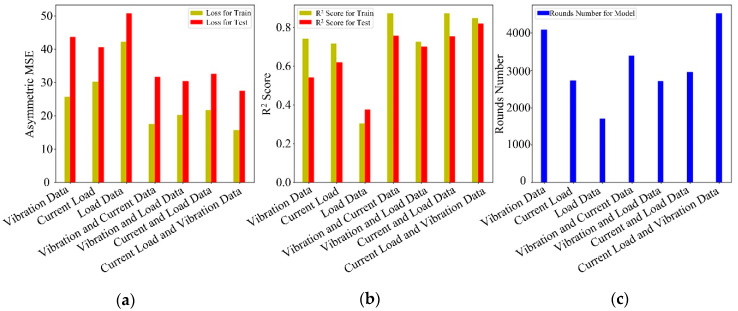
Comparison chart of different evaluations. (**a**) Asymmetric Loss. (**b**) R^2^ Score. (**c**) Boosting Rounds.

In Table 3, it can be seen that asymmetric loss does not show good results on both the test set and the training set when using the single data type, and the best fit of the single type of data is only 0.6184. In the case of using two data types, we can clearly see that the combination of any data type is greatly improved compared to the previous single data type. By contrast, when using three data types, the current signal, load signal and vibration signal can be effectively combined to better characterize the change of cutting force. Although the convergence speed of the model slowed down, there was a significant improvement in the generalization ability of the model (27.472 in asymmetric loss (test) and 0.8176 in R^2^ Score (test)). The result shows that the multi-information fusion technology implemented by three types of data combination can effectively reduce the error size of the model and improve the generalization ability of the model and better predict the target value. For data combinations, we wanted to perform common feature extraction on different data signals and we did not want to spend too much time at this step. This step focuses more on the effect changes brought about by the use of data types, rather than the application of data feature extraction methods. In order to more intuitively compare the error size and generalization ability of the model, Figure 7 shows a comparison chart of different evaluations.

### 3.3. The Effectiveness of SWM-CA

Feature extraction in Section 3.2 was performed over the entire time interval, which will cause some of the local key features to be ignored due to excessive time granularity. In order to solve this problem, this paper used the sliding windows method to perform small-time fine-grained interception and increment raw data in units of unit time and extracted the feature, and then was taken as a feature vector into the clustering algorithm. The Silhouette coefficient determines the optimal number of clusters.

(7) $$s(i)=\frac{\frac{a}{a+b+c}-\frac{1}{k}\sum_{i=1}^{k}\underset{j\neq i}{\max}\left(\frac{avg(C_{i})+avg(C_{j})}{d_{cen}(C_{i},C_{j})}\right)}{\max\left\{\frac{1}{k}\sum_{i=1}^{k}\underset{j\neq i}{\max}\left(\frac{avg(C_{i})+avg(C_{j})}{d_{cen}(C_{i},C_{j})}\right),\frac{a}{a+b+c}\right\}}$$

where *C_i_*, *C_j_*, *k* is Davies–Bouldin index params; *a*, *b*, *c* is Jaccard coefficient params.

**Figure 8:**
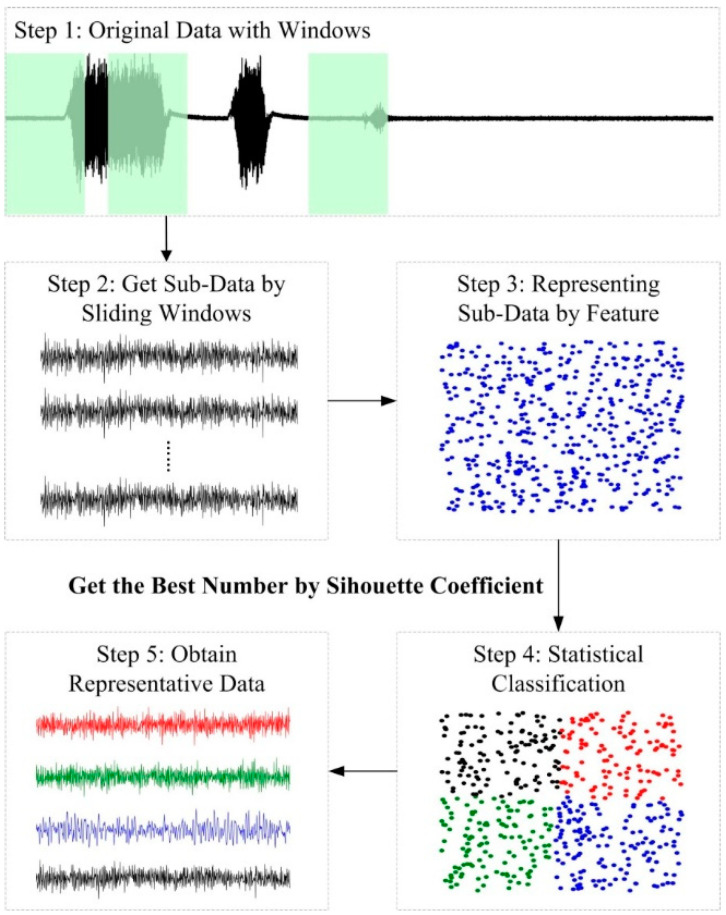
The flowchart of sliding windows method clustering algorithm (SWM-CA).

The closer the distance between the samples are in the cluster, the farther the distance between the samples is, namely the smaller the DB index is, the larger the Jaccard is, the larger the average contour coefficient is, and the better the clustering effect is, and so a best cluster number *k* = 4 is obtained. The next step it to set the number of classification categories, and then obtain the subdata groups of the four groups of categories, and finally add all the subdata segment features to the original feature matrix. This is transformed into a small-time fine-grained feature representation which uses the density center data of these four types of data groups as a substitute. Figure 8 shows the flowchart of SWM-CA.

In order to verify effect of the proposed SWM-CA, two groups of comparative experiments were conducted. That is, under the condition of using the same model, the residual distribution was compared between the model using SWM-CA and the model without SWM-CA, and the distribution range and distribution law were are observed.

**Figure 9:**
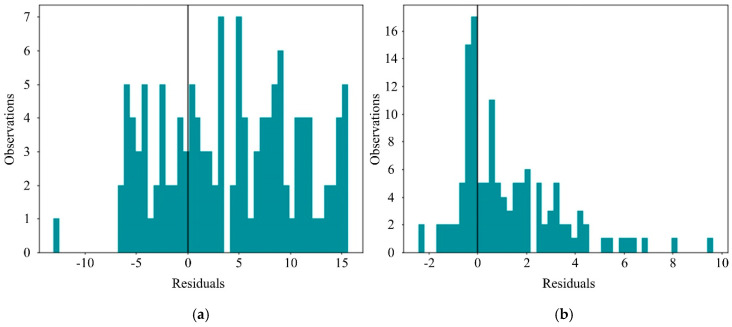
Error histograms of predictions from different group. (**a**) LightGBM with raw feature. (**b**) LightGBM with secondary extraction feature.

Figure 9 shows the result of the comparison. Since we imposed penalties on negative residuals, the data distribution is unequal on both sides. The residuals used in the original data features have a wide range of distributions. By contrast, the residual distribution range of the secondary extraction feature using SWM-CA is narrowed, and the residual is more concentrated on the positive side, which ensures that the absolute error is reduced, further reducing the harm in real production. The result shows that: (1) the secondary feature extraction using SWM-CA can effectively cover small-time fine-grained features; (2) SWM-CA can effectively expand features and enhance the generalization ability of the model.

### 3.4. Comparative Experiments of the RUL Predictive Model

In order to verify the effect of the improved LightGBM, comparisons were made using an improved machine learning model (*p*-LightGBM) and an unimproved machine learning model. Table 4 shows the result of the comparison. First of all, for the data part, we used the dataset that had not been used by the model—that is, the full-life data collected by the two ball-end mills. Through such dataset verification, we could verify the generalization ability of the model because, compared to our previous training set, this part of the data and the data used may have the same distribution as the previous dataset. However, since we are not using this dataset, we can use it to generalize the validation model. The improved model exhibits a better performance, although there is a slight loss in the fit and loss on the training set, but overall, it has better adaptability to unknown data. It has a faster convergence speed and is more in line with the actual industrial demand forecast results.

In order to more comprehensively observe the performance of the improved model before and, after the improvement, explain how well the model performs in sample intervals of different sizes, comparisons were made by using sample test sets of different sizes (20% sample and 100% sample).

**Figure 10:**
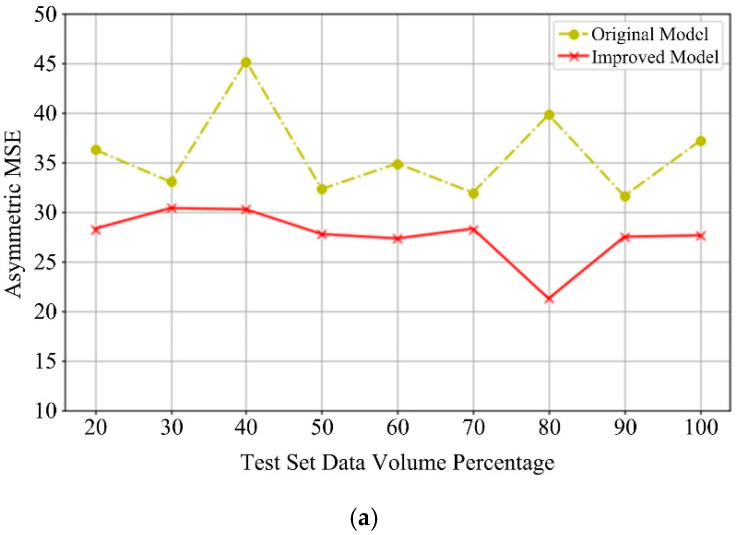

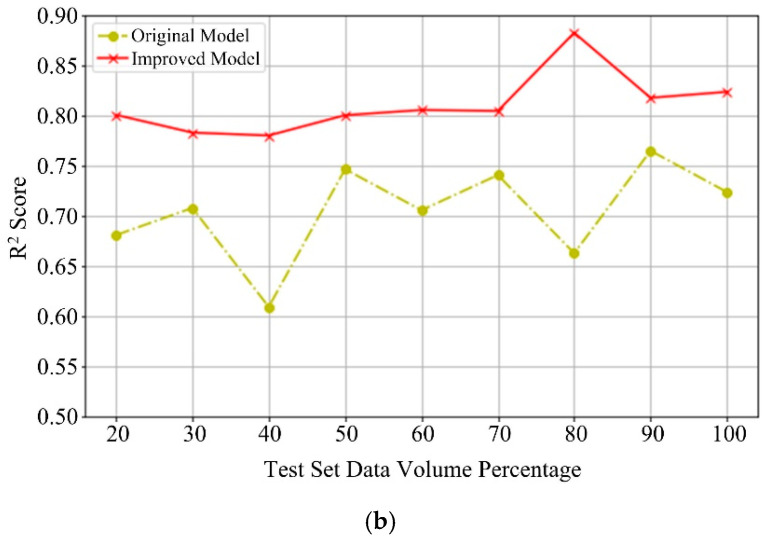
Comparison results of different evaluations under different sample. (**a**) Asymmetric Loss. (**b**) R^2^ Score.

Figure 10 shows the result of the comparison. With the improved LightGBM, the error and accuracy will fluctuate slightly as the sample size changes. However, overall, it is still better than the state before the improvement. The result shows that, considering the strategy of multi-information fusion, combining different data types, using the SWM-CA to unsupervise the data and extract the features twice, and adopting the improved LightGBM model, can maintain better generalization ability and prediction accuracy.

## 4. Conclusions

This paper presents a LightGBM-based approach to RUL prediction related to process and machining state. We establish a CLF considering the importance coefficient p to realize the self-adaptive adjustment of the imbalance between the two sides of the risk, reduce the loss in the opposite direction to the industrial expectation, achieve a secondary correction of the loss and match the industrial production practice. In the data preprocessing stage, a multi-information fusion strategy was adopted to reduce the error of the model, improving the confidence of features and improving the generalization ability of the model. At the same time, the unsupervised analysis of the subsamples after sliding windows method segmentation was performed by a clustering algorithm, which not only takes the small-time fine-grained features into account but also avoids the curse of dimensionality of features. The effectiveness of the proposed method was verified by an experimental comparison evaluation. Based on data evaluation results, it can be observed that considering the multi-information fusion strategy and the secondary extraction feature using the sliding windows method and clustering algorithm, the improved LightGBM model was used for RUL prediction, which is more in line with industrial needs and has better generalization ability and prediction accuracy.

For further research in the future, we will not be limited to the GBDT model, but will focus more on the application of attention technology. We will find a way to combine the actual industrial pain points and technical advantages for further exploration. There is no doubt that this method can not only be applied to cutting tools, but can also be transplanted to other objects. Therefore, future research will carry out further experiments and research on the universality of this method.

## Figures and Tables

**Table 1:** Model fitting condition under different comparisons.

Model Setting	Boosting Rounds	MSE (Train)	Asymmetric Loss (Train)	Asymmetric Loss (Test)
LightGBM default	100	0.236246	0.628296	1.31852
LightGBM with custom loss	100	0.330155	0.27638	0.819872
LightGBM with eraly stopping	780	0.137639	0.0531724	0.783725
LightGBM with early stopping and custom loss	1848	0.162248	0.0136494	0.868132
LightGBM with early stopping, custom loss and custom validation loss	241	0.22839	0.13002	0.740384

**Table 2:** Compare the feature set used for verification.

Features	Equations
Root mean square *x_rms_*	$x_{rms}={\left(\frac{1}{N}\sum_{i=1}^{N}x_{i}^{2}\right)}^{1/2}$
Square root amplitude *x_sra_*	$x_{sra}={\left(\frac{1}{N}\sum_{i=1}^{N}\sqrt{\left|x_{i}\right|}\right)}^{2}$
Kurtosis value *x_kv_*	$x_{kv}=\frac{1}{N}\sum_{i=1}^{N}{\left(\frac{x_{i}-\bar{x}}{\sigma }\right)}^{4}$
Skewness value *x_sv_*	$x_{sv}=\frac{1}{N}\sum_{i=1}^{N}{\left(\frac{x_{i}-\bar{x}}{\sigma }\right)}^{3}$
Peak to peak value *x_ppv_*	$x_{ppv}=\max(x_{i})-min(x_{i})$
Crest factor *x_cf_*	$x_{cf}=\max(\left|x_{i}\right|)/{\left(\frac{1}{N}\sum_{i=1}^{N}x_{i}^{2}\right)}^{1/2}$
Impusle factor *x_if_*	$x_{if}=\max(\left|x_{i}\right|)/\frac{1}{N}\sum_{i=1}^{N}\left|x_{i}\right|$
Clearance factor *x_CF_*	$x_{CF}=\max(\left|x_{i}\right|)/{\left(\frac{1}{N}\sum_{i=1}^{N}\sqrt{\left|x_{i}\right|}\right)}^{2}$
Center of gravity frequency	$FC=\int_{0}fS(f)df/\int_{0}^{+\infty }S(f)df$
Mean square frequency	$MSF=\int_{0}^{+\infty }f^{2}S(f)df/\int_{0}^{+\infty }S(f)df$
Root mean square frequency	$RMSF=\sqrt{MSF}$
Variance of frequency	$VF=\int_{0}^{+\infty }{\left(f-FC\right)}^{2}S(f)df/\int_{0}^{+\infty }S(f)df$

**Table 3:** Predictions for different types and different combinations data types.

Model Setting	Boosting Rounds	Asymmetric Loss (Test)	Asymmetric Loss (Train)	R^2^ Score (Test)	R^2^ Score (Train)
LightGBM with Vibration Data	4083	43.616	25.629	0.5407	0.7411
LightGBM with Current Data	2713	40.591	30.194	0.6184	0.7146
LightGBM with Load Data	1707	50.859	42.304	0.3748	0.3030
LightGBM with Vibration and Current Data	3384	31.744	17.579	0.7561	0.8718
LightGBM with Vibration and Load Data	2699	30.360	20.254	0.6987	0.7237
LightGBM with Current and Load Data	2940	32.607	21.716	0.7545	0.8732
LightGBM with Current, Load and Vibration Data	4522	27.472	15.615	0.8176	0.8465

**Table 4:** Predictions for different types and different combinations data types.

Model Setting	Boosting Rounds	Asymmetric Loss (Test)	Asymmetric Loss (Train)	R2 Score (Test)	R2 Score (Train)
LightGBM with Default Data Feature	4522	27.472	15.615	0.81760	0.8465
*p*-LightGBM with New Data Feature	4227	24.383	16.542	0.8268	0.8357
